# Effects of Intraoperative Dexamethasone and Ondansetron on Postoperative Nausea and Vomiting in Microvascular Decompression Surgery: A Randomized Controlled Study

**DOI:** 10.1155/2018/6297362

**Published:** 2018-11-11

**Authors:** Cattleya Thongrong, Patitha Chullabodhi, Pornthep Kasemsiri, Amnat Kitkhuandee, Narin Plailaharn, Lumyai Sabangban, Thirada Jimarsa

**Affiliations:** ^1^Department of Anesthesiology, Faculty of Medicine, Khon Kaen University, Khon Kaen, Thailand; ^2^Division of Skull Base Surgery, Department of Otorhinolaryngology, Faculty of Medicine, Khon Kaen University, Khon Kaen, Thailand; ^3^Division of Neurosurgery, Department of Surgery, Faculty of Medicine, Khon Kaen University, Khon Kaen, Thailand

## Abstract

**Background:**

Postoperative nausea and vomiting (PONV) is a common problem and may lead to catastrophic complications, especially in neurosurgical cases. The aim of this study was to evaluate the effects of dexamethasone and ondansetron for preventing PONV in patients who underwent microvascular decompression (MVD) surgery.

**Methods:**

A prospective, double-blinded, randomized control trial was conducted with 54 patients who underwent MVD. Patients were allocated into two groups. The study group (Gr. D) received intraoperative dexamethasone 4 mg iv and ondansetron 4 mg iv, whereas the control group (Gr. N) received placebo (0.9% normal saline 1 ml iv and 0.9% normal saline 2 ml iv). The incidence and severity of PONV were observed at 1, 2, 4, and 24 hr postsurgery.

**Results:**

At 1, 2, 4, and 24 hr postsurgery, Gr. D had a lower incidence (7.4%, 11.1%, 29.6%, and 66.7%) and lower severity of PONV than Gr. N (18.5%, 29.6%, 37.0%, and 81.5% at 1, 2, 4, and 24 hr; *p* > 0.05). The requirement for antiemetic drugs was not significantly different between the groups (*p* > 0.05).

**Conclusion:**

Administration of dexamethasone and ondansetron 4 mg seemed to decrease the incidence of PONV in the first 24 hours but not significantly. Therefore, further studies are to be carried out by escalating either dexamethasone dose or the dose of ondansetron or both.

## 1. Introduction

Postoperative nausea and vomiting (PONV) is a common complication. The consequences of PONV are unfavorable and can prolong the length of a hospital stay because of complications, such as, discomfort, aspiration pneumonia, bleeding from the surgical wound, and wound dehiscence [1]. PONV can be influenced by multiple factors, including sex, age, type of anesthesia, type of surgery, and intraoperative analgesic drugs [1, 2].

Approximately 40–70% of patients who undergo neurosurgery suffer from PONV in the first 24 hours [2–5]. But the incidence of PONV following microvascular decompression (MVD) surgery hovers around 70% [5]. Such high incidence of PONV is due to the close proximity of the operating field to the chemoreceptor trigger zone or the area postrema (vomiting center). PONV in a neurosurgical operation may induce increased intracranial pressure or even cause life-threatening brain herniation and death [6–8].

Dexamethasone and ondansetron are commonly used for prophylaxis of PONV [9–13] due to their negligible adverse effects. They are prescribed in several surgical procedures, including laparoscopic cholecystectomy, obstetrical-gynecological surgery, and craniotomy. Literature search did not reveal any clinical trial on the intraoperative use of dexamethasone and ondansetron for prevention of PONV in patients following MVD. To address the above issue, a double-blinded, randomized controlled clinical study was carried out.

## 2. Methods

This prospective, randomized double-blinded controlled trial was conducted at Srinagarind Hospital, Khon Kaen, Thailand, from August 7, 2014, to February 16, 2016. Patients who were scheduled for MVD of the trigeminal nerve root were enrolled. The study group (Gr. D) received intraoperative dexamethasone 4 mg and ondansetron 4 mg, whereas the control group (Gr. N) received placebo (0.9% normal saline 1 ml and 0.9% normal saline 2 ml). Patients of either gender who were at least 18 years old, those who had a physical status classification of I to III according to the American Society of Anesthesiologists, and those who had a body mass index of 18–35 kg/m^2^ were included. The exclusion criteria were patients on long-term administration of dexamethasone or ondansetron, those with a history of allergic reactions to dexamethasone or ondansetron, those who had undergone antiemetic therapy within 24 hr before surgery, those with underlying liver or renal failure, those who were pregnant, or those who had undergone emergency surgery. This research was approved by the Human Research Ethics Committee of Khon Kaen University (HE571218). In addition, this study was registered with the ClinicalTrial.gov (NCT03685032). The sample size was calculated on the basis of the incidence of PONV following MVD in our hospital database. An 80% different incidence of PONV was considered as a clinically relevant difference. Considering a significance level of 0.05 and power of 0.8, we needed 27 patients for each group.

Fifty-four patients were allocated into 2 groups (Gr. D 27 patients and Gr. N 27 patients) by computer-generated randomization (block of four). All patients received 100% oxygen for 3 minutes before induction with fentanyl 1–1.5 mcg/kg, 2% xylocaine 1–1.5 mg/kg, propofol 1.5–2 mg/kg, and cis-atracurium 0.15 mg/kg. Subsequently, endotracheal tubes were intubated and connected to the anesthetic circuit with controlled ventilation. The ventilation settings were a respiratory rate of 12 times per minute, a tidal volume of 6–8 ml/kg, and an end-tidal CO_2_ of 30–35 mmHg. Ventilation was assisted with 2% sevoflurane in an adjusted oxygen airflow of 1:1 liter per minute. After patients received general anesthesia, a sequentially numbered opaque sealed envelope was opened. Gr. D was administered 4 mg of dexamethasone in 1 ml iv, and Gr. N received normal saline 1 ml iv. At the end of the operation when suturing the dura mater, Gr. D received ondansetron 4 mg in 2 ml iv while Gr. N received normal saline 2 ml iv. The study drugs based on a sequentially numbered list were prepared in the same way. These drugs had similar characteristics, including clear color and no observable particles, and were loaded into syringes labeled for each group. The attending anesthesiologists, anesthetic nurses, and ward nurses, as well as the patients were blinded to the computer-generated randomization lists.

After completing the operation, the patients were evaluated for the incidence and severity of PONV and a pain score at 1 and 2 hr in the postoperative period in the recovery room and at 4 and 24 hr in the ward by anesthetic nurses. The patients could request antiemetic and opioid analgesic medications, and the doses were recorded as well as the level of satisfactory reduction of PONV (0 = no symptoms; 1 = mild: few symptoms and not requiring treatment; 2 = moderate: presented symptoms and needed ondansetron 8 mg iv; 3 = severe: persisted symptoms after received ondansetron 8 mg iv and needed re-administration of ondansetron 8 mg iv). If symptoms still persist after re-administration of ondansetron, metoclopramide 10 mg iv will be administrated. The intensity of postoperative pain was measured with a numeric rating scale (NRS: 0 = no pain and 10 = severe pain). An independent *t*-test, chi-squared test, and Fisher's exact test were used as appropriate for data analysis. *P* < 0.05 was considered statistically significant.

## 3. Results

Fifty-four patients were allocated into two groups (Gr. N and Gr. D), and there were no differences in the demographic baseline data (*p* > 0.05) (Table 1). Important confounding factors included the consumption of opioids, which showed no statistically significant differences in the postoperative period (*p* > 0.05) (Table 2). At 1, 2, 4, and 24 hr in the postoperative period, Gr. D had a lower incidence of PONV than Gr. N (*p* > 0.05) (Table 3). The requirement for antiemetic drugs did not differ significantly between the groups (*p* > 0.05) (Table 4).

## 4. Discussion

PONV is a common serious problem, especially in neurosurgical patients, because of the morbidity and mortality complications resulting from elevated intracranial and arterial pressure [14]. The incidence of PONV following MVD is high. Meng and Quinlan [8] and Vengkatraghavan et al. [15] reported an incidence of 60% within the first 24 hr. Joo et al. [5] found that 69.7% of patients undergoing MVD developed PONV. Additionally, Ha et al. [16] found the high incidences despite the use of antiemetic prophylaxis. The ramosetron prophylaxis allowed the incidences of nausea of 87.1% and vomiting of 51.6%, whereas ondansetron prophylaxis allowed the incidence of nausea of 93.6% and vomiting of 61.3%.

In the present study, the incidence of PONV was similar to that in other studies. It was found that patients had a higher incidence of PONV following postoperative duration. In Gr. N, incidences of 18.5%, 29.6%, 37.0%, and 81.5% were observed at 1, 2, 4, and 24 hr, respectively. This trend of incidence was similar in the other group that was administered dexamethasone 4 mg and ondansetron 4 mg. For this group, the incidences were reported to be 7.4%, 11.1%, 29.6%, and 66.7% at 1, 2, 4, and 24 hr, respectively. Although the administration of dexamethasone and ondansetron was found to decrease the incidence of PONV, this decrease was not significantly different (*p* > 0.05). However, these results were different from the findings of Kathirvel et al. [10], who compared dexamethasone 4 mg iv alone with dexamethasone 4 mg iv and ondansetron 4 mg iv in patients who underwent elective craniotomy for resection of various intracranial tumors and vascular lesions. They found that the incidence of postoperative emesis was significantly reduced in patients who received dexamethasone and ondansetron (11%) compared with those who received dexamethasone alone (39%) (*p* < 0.001). Regarding our results, administration of dexamethasone 4 mg and ondansetron 4 mg may not be statistically significant effective therapy in MVD because this operation is a high probability of PONV. Furthermore, the efficacy of another antiemetic prophylactic drug (i.e., ramosetron) in MVD was investigated. There was no difference in preventive efficacy between ramosetron and ondansetron [16]. Currently, antiemetic prophylaxis against PONV is a lack of sufficient emphasis in MVD. However, antiemetic prophylaxis in the infratentorial craniotomy reported that a larger dose of ondansetron 8 mg at the time of wound closure decreased the incidence of PONV [17]. Therefore, further studies should be conducted to investigate this hypothesis in MVD with a larger sample size and escalating either doses of dexamethasone or ondansetron or both.

Furthermore, the incidence of PONV was higher at 24 hr after surgery, whereas a lower incidence of PONV at 1 hr, 2 hr, and 4 hr was found. This result may have been affected by propofol. Several previous studies reported that propofol alone reduces PONV. Sneyd et al. [18], who analyzed prospective randomized comparator studies, suggested that there was a reduction in PONV following maintenance of anesthesia with propofol compared with inhalational agents. They found a significantly lower incidence of PONV in the propofol group. Regarding the severity of PONV, the present study found that most patients had mild PONV, and half of these patients needed antiemetic rescue, especially at 24 hr. Unfortunately, the differences between using antiemetic drugs between the two groups were not statistically significant (*p* > 0.05).

There are several possible limitations of this study. First, doses of dexamethasone and ondansetron were too small to provide the antiemetic prophylactic effect following MVD because this operation, relative to other neurosurgical procedures, has a potentially high risk for PONV. Second, it was overestimated that dexamethasone 4 mg and ondansetron 4 mg could reduce the incidence of PONV from a control group by 80%. This estimation led to a small sample size with a low power of preventive emesis effect.

## 5. Conclusion

Administration of dexamethasone 4 mg and ondansetron 4 mg as intraoperative medications for patients who underwent MVD seemed to decrease the incidence of PONV but did not reach statistical significance. Therefore, further studies should be developed by escalating either dexamethasone dose or ondansetron dose or both.

## Figures and Tables

**Table 1:** Demographic data.

	Gr. N (*n*=27)	Gr. D (*n*=27)	*p* value
*Gender*			
Male (%)	8 (29.6)	9 (33.3)	0.77
Female (%)	19 (70.4)	18 (66.7)	
Age (yr, mean)	35–69 (56.07)	29–74 (55.62)	0.868
Weight (kg, mean)	44–97 (64.70)	32–90 (61.11)	0.357
Height (cm, mean)	140–175 (159.92)	145–175 (159.48)	0.824

*ASA class*			
I (%)	11 (40.7)	14 (51.9)	0.373
II (%)	16 (59.3)	13 (48.1)	
III (%)	0	0	

*Age < 50 yr*			
Yes (%)	6 (22.2)	9 (33.3)	0.362
No (%)	21 (77.8)	18 (66.7)	

*Smoking*			
Yes (%)	3 (11.1)	4 (14.8)	0.500
No (%)	24 (88.9)	23 (85.2)	

*Motion sickness*			
Yes (%)	4 (14.8)	4 (14.8)	0.648
No (%)	23 (85.2)	23 (85.2)	

*History of PONV*			
Yes (%)	2 (7.4)	5 (18.5)	0.21
No (%)	25 (92.6)	22 (81.5)	

*Duration of surgery (min, mean)*	55–150 (103.33)	55–145 (98.89)	0.452

*Fentanyl used intraoperative (mcg, mean)*	100–200 (143.51)	100–225 (136.11)	0.365

**Table 2:** Pain score and opioid analgesics consumed in the 24 hr postoperative period.

	Gr. N (*n*=27)	Gr. D (*n*=27)	*p* value
*Postoperative pain score (mean NRS)*			
At 1 hr	4.6 (95% CI: 0–10)	4.0 (95% CI: 0–8)	0.725
At 2 hr	3.2 (95% CI: 0–8)	2.9 (95% CI: 0–7)	0.640
At 4 hr	2.7 (95% CI: 0–6)	2.6 (95% CI: 0–6)	0.562
At 24 hr	4.4 (95% CI: 0–10)	4.0 (95% CI: 0–8)	0.691

*Mean opioid consumption*			
*Fentanyl (mcg, mean)*			
At 1 hr	31.5 (95% CI: 0–75)	28.7 (95% CI: 0–100)	0.738
At 2 hr	5.6 (95% CI: 0–50)	3.7 (95% CI: 0–25)	0.539
At 4 hr	1.9 (95% CI: 0–25)	0.9 (95% CI: 0–25)	0.561
At 24 hr	0	0	0

*Morphine (mg, mean)*			
At 24 hr	3.11 (95% CI: 0–6)	2.44 (95% CI: 0–6)	0.245

**Table 3:** Incidence of postoperative nausea and vomiting and its severity in the 24 hr postoperative period.

Postoperative time		PONV and grading (*n*)				*p* value
		Mild	Moderate	Severe	Total, *n* (%)	
At 1 hr	Gr. N (*n*=27)	1 (95% CI: 0.7–18.3)	3 (95% CI: 3.9–28.1)	1 (95% CI: 0.7–18.3)	5 (18.5) (95% CI: 8.2–36.7)	0.669
	Gr. D (*n*=27)	1 (95% CI: 0.7–18.3)	1 (95% CI: 0.7–18.3)	0	2 (7.4) (95% CI: 2.1–23.3)	

At 2 hr	Gr. N (*n*=27)	7 (95% CI: 13.2–44.7)	0	0	(95% CI: 13.2–44.7)	0.175
	Gr. D (*n*=27)	3 (95% CI: 3.9–28.1)	0	0	3 (11.1) (95% CI: 3.9–28.1)	

At 4 hr	Gr. N (*n*=27)	10 (95% CI: 21.5–55.8)	0	0	(95% CI: 21.5–55.8)	0.372
	Gr. D (*n*=27)	6 (95% CI: 10.6–40.7)	1 (95% CI: 0.7–18.3)	1 (95% CI: 0.7–18.3)	8 (29.6) (95% CI: 15.9–48.5)	

At 24 hr	Gr. N (*n*=27)	16 (95% CI: 40.7–75.5)	6 (95% CI: 10.6–40.7)	0	(95% CI: 63.3–91.8)	0.202
	Gr. D (*n*=27)	10 (95% CI: 21.5–55.8)	6 (95% CI: 10.6–40.7)	2 (95% CI: 2.1–23.3)	18 (66.7) (95% CI: 47.8–81.4)	

**Table 4:** Antiemetics used in the 24-hr postoperative period.

			Postoperative period (*n*)			
			1 hr	2 hr	4 hr	24 hr
N (*n*=27)	Ondansetron	8 mg	4 (95% CI: 5.9–32.5)	0	0	3 (95% CI: 3.9–28.1)
	Metoclopramide	10 mg	0	0	0	4 (95% CI: 5.9–32.5)

D (*n*=27)	Ondansetron	8 mg	1 (95% CI: 0.6–18.3)	0	2 (95% CI: 2.1–23.4)	5 (95% CI: 8.2–36.7)
	Metoclopramide	10 mg	0	0	0	3 (95% CI: 3.9–28.1)

*p* value	Ondansetron		0.167	—	0.161	0.453
	Metoclopramide		—	—	—	0.731

## Data Availability

The data used to support the findings of this study are included within the article.

## Abbreviations

PONV: postoperative nausea and vomiting
MVD: microvascular decompression surgery
iv: intravenous.
